# Preoperative HE4 and ROMA values do not improve the CA125 diagnostic value for borderline tumors of the ovary (BOT) – a study of the TOC Consortium

**DOI:** 10.1186/1757-2215-7-49

**Published:** 2014-05-07

**Authors:** Elena Ioana Braicu, Toon Van Gorp, Mani Nassir, Rolf Richter, Radoslav Chekerov, Khayal Gasimli, Dirk Timmerman, Ignace Vergote, Jalid Sehouli

**Affiliations:** 1 Department of Gynecology, Campus Virchow Clinic, Charité Medical University Berlin, Augustenburger Platz 1, Berlin 13353, Germany; 2 Department of Obstetrics and Gynecology, Maastricht University Medical Centre, PO Box 5800, Maastricht 6202 AZ, The Netherlands; 3 Department of Obstetrics and Gynecology, University Hospital KU Leuven, Herestraat 49, Leuven 3000, Belgium

**Keywords:** HE4, CA125, ROMA, Borderline ovarian tumors, Invasive implants

## Abstract

**Background:**

Borderline tumors of the ovary (BOT) are a distinct entity of ovarian tumors, characterized by lack of stromal invasion. Recent studies postulated that the presence of invasive implants, incomplete staging, fertility sparing surgery and residual tumor after surgery are major prognostic factors for BOT. There are no biomarkers that can predict BOT or the presence of invasive implants.

**Objective:**

The aim of our study was to assess the value of CA125 and HE4 alone, or within ROMA score for detecting BOT, and for predicting the presence of invasive implants.

**Methods:**

Retrospective, monocentric study on 167 women diagnosed with BOT or benign ovarian masses. Serum HE4, CA125 levels and ROMA were assessed preoperatively. Due to low number of BOT with invasive implants, we performed an unmatched analysis (consecutive patients) and a matched analysis (according to age and histology) to compare BOT with invasive implants, BOT without invasive implants and benign disease.

**Results:**

There were no significant differences in the HE4 and CA125 expressions in the three groups of patients (p = 0.984 and p = 0.141, respectively). The ROC analysis showed that CA125 alone is superior to ROMA and HE4 in discriminating patients with BOT with invasive implants from patients with benign diseases and BOT without invasive implants. A newly established score, ROMABOT, did not perform better than ROMA. The analysis of the matched groups revealed similar results as the analysis of all samples.

**Conclusions:**

Both HE4 and CA125 are not reliable biomarkers for the diagnosis of BOT or for predicting the presence of invasive implants.

## Introduction

Borderline tumors of the ovary (BOT) are an independent entity of ovarian tumors, being characterized by the presence of cellular proliferation and nuclear atypia without any destructive stromal invasion [1,2]. However, they may present with microinvasion, lymph node implants, and extra-ovarian implants that can be either non-invasive or invasive [3]. BOT are divided histologically into serous (53.3%), mucinous (42.5%) and other (4.2%) less common subtypes, including endometrioid, clear cell, transitional cell and Brenner tumors [4,5].

BOT affect younger women with one third of the patients being under 40 years of age at time of initial diagnosis [6]. In contrast to ovarian cancer, BOT are diagnosed in earlier FIGO stages and show generally a favorable prognosis [2,7,8]. The standard of care for BOT still comprises bilateral oophorectomy together with comprehensive surgical staging, consisting of peritoneal biopsies, infracolic omentectomy, peritoneal washings and removal of all macroscopic peritoneal implants [2,9-11]. Despite such radical surgery, up to 10-30% of the patients will develop late recurrence, with around 30% of them being diagnosed with invasive ovarian cancer [12]. A recent study conducted by Du Bois et al. underlined that FIGO stage, quality of surgical treatment and histological examination are the most important prognostic factors regarding relapse rate and outcome in BOT patients [12]. Therefore a comprehensive surgical staging is needed in order to detect the presence of invasive extra-ovarian implants [12].

To date, the cancer antigen 125 (CA125) is the most commonly used tumor marker in the evaluation and clinical management of an ovarian mass, but since it has a low specificity, especially in premenopausal women [13,14], the search for complementary biomarkers is pivotal. Human Epididymis Protein 4 (HE4), a whey-acid protein first isolated in the epithelium of human epididymis and in epithelial cells of the respiratory system as well as in the female reproductive tract [15-17], offers superior specificity in the differentiation of benign and malignant adnexal masses in premenopausal women compared to CA125 [18]. The Risk of Ovarian Malignancy Algorithm (ROMA) was developed by Moore et al. and combines CA125 and HE4 serum levels and the menopausal status of a patient with suspicious pelvic mass, thus stratifying patients into high and low risk groups for having a malignant ovarian lesion [19].

Until now, there are no clinical or biomarkers to predict the presence of invasive implants in BOT. In this study we analyzed the value of individual CA125, HE4 and the combination of both markers in the ROMA score for detecting BOT, and for predicting the presence of invasive implants.

## Methods

In the current study samples from a total of 167 patients with either benign gynecological diseases or BOT with or BOT without invasive peritoneal implants were collected prospectively, within the Tumor Bank Ovarian Cancer project (TOC).

All patients received surgical treatment in our comprehensive center for ovarian cancer treatment at the Department of Gynecology, Virchow Campus Clinic, Charité Medical University of Berlin.

Written informed consent was obtained before the collection of serum samples. Ethical approval for this study was provided by the ethics committee at the Charité Medical University of Berlin (EK207/2003).

### Collection of serum samples

Serum samples were obtained before surgery. After centrifugation and aliquotation into cryovials, samples were frozen at −80°C until further usage.

### HE4 ELISA

HE4 concentrations in serum were measured using the HE4 EIA assay (Fujirebio Diagnostics AB, Gothenburg, Sweden). Each sample was analyzed in duplicate. The appropriate controls were within the ranges provided by the manufacturer.

### CA 125

CA125 was determined in serum during the routine analysis by using Roche Kits.

### ROMA

To stratify pre- and postmenopausal patients into either low or high-risk groups for the presence of a malignant pelvic mass, we used the following equations in order to calculate the ROMA score (as introduced by Moore et al.) [19,20]:

$$\begin{array}{l} 1.\;\mathrm{Premenopausal}:\mathrm{predictive}\;\mathrm{index}\;\left(\mathrm{PI}\right)\\ \;=-12.0+2.38*\mathrm{LN}\;\left[\mathrm{HE}4\right]+0.0626*\mathrm{LN}\left[\mathrm{CA}125\right]\\ 2.\;\mathrm{Postmenopausal}:\mathrm{predictive}\;\mathrm{index}\;\left(\mathrm{PI}\right)\\ \;=-8.09+1.04*\mathrm{LN}\;\left[\mathrm{HE}4\right]+0.732*\\ \;\mathrm{LN}\;\left[\mathrm{CA}125\right]\mathrm{ROMA}\;\mathrm{score}\;\left(\%\right)\\ \;=\exp\left(\mathrm{PI}\right)/\left[1+\exp\left(\mathrm{PI}\right)\right]*100. \end{array}$$

Therefore the ROMA Index is mainly determined by HE4 in premenopausal status and by both biomarkers in postmenopausal patients.

### Statistical analysis

The clinical data were collected and entered in a SPSS database. All analyses were performed with PASW 21.0 (SPSS Inc., Chicago).

Furthermore, we identified a cohort of 14 BOT patients with invasive implants that were matched by histology and correspondent age in order to minimize bias with another 14 patients presenting with non-invasive implants. In addition, every BOT patient with invasive implants was matched with a total of 4 age-correspondent patients with benign ovarian masses.

Continuous variables were compared with the Kruskal-Wallis test; p-values for pairwise comparisons were presented without adjustment for multiple testing. Receiver operator characteristics (ROC) curve analysis was performed to evaluate the predictive accuracy of HE4 and CA125 expression for discriminating patients with borderline or benign tumors from patients with BOT and invasive peritoneal implants.

Adjusted hazard ratios (HR) and 95% CI for prognostic factors were calculated. A two-tailed p-value < 0.05 was considered as statistically significant.

## Results

### Baseline characteristics

We enrolled 167 patients with BOT and benign diseases. A total of 104 patients were diagnosed with benign masses, including ovarian fibrothecoma and uterine leiomyomata. Forty-eight patients presented with BOT without invasive implants, and 15 patients with invasive implants. The menopausal status of all patients was assessed stratifying them into either being premenopausal (N = 104) or postmenopausal (N = 61), respectively. Two patients were excluded since the menopausal status was unclear. Patient characteristics are presented in Table 1.

**Table 1:** Patient characteristics

**Parameter**	**N**	**Median**	**Range**
**Age (years)**			
Whole collective	167	47	16-87
Benign diseases	104	48	19-86
BOT without InvImp	48	44	16-83
BOT with InvImp	15	55	25-87
**Preoperative HE4 (pmol/L)**			
Benign diseases	104	50	20-1410
BOT without InvImp	48	52	29-2135
BOT with InvImp	15	74	35-461
**Preoperative CA125 (IU/ml)**			
Benign diseases	104	21	5-334
Non-invasive BOT	48	25	3-725
Invasive BOT	15	63	12-259
**Histological type of BOT**	**N (without InvImp)**	**N (with InvImp)**	**N (total)**
Serous	29	10	39
Mucinous	18	4	22
Endometrioid	0	1	1
Mixed	1	0	1
**Types of benign diseases**	**N**	**%**	
Benign ovarian lesions	48	49.92	
Ovarian thecofibroma	2	2.08	
Leiomyomata	55	57.2	

*Abbreviations*: BOT: borderline ovarian tumors; InvImp: invasive implants; HE4: human epididymis protein 4; CA125: cancer antigen 125.

### HE4 and CA125 serum concentrations in premenopausal patients

Median HE4 expression in serum of premenopausal patients was 49 pM (range: 20-1410 pM) in benign diseases, 37 pM (range: 35–94.6 pM) and 45.1 pM (range: 29-2135 pM) in BOT patients with and without invasive implants, respectively.

Preoperative median CA125 serum concentrations in premenopausal patients were 21 U/ml (range: 7-251 U/ml) in benign diseases, 32 U/ml (range: 19-232 U/ml) and 24.5 U/ml (range: 3-725 U/ml) in BOT with and without invasive implants, respectively.

Among premenopausal patients there was no significant differences in the HE4 (p = 0.984) and CA125 (p = 0.141) expressions in the three groups of patients.

The AUC for HE4 and CA125 were 0.515 (95% CI. 0.212-0.818) and 0.710 (95% CI 0.512-0.908), respectively.

Furthermore, we evaluated the role of ROMA in predicting BOT and presence of invasive implants. The AUC for ROMA algorithm was 0.521 (95% CI 0.223-0.820).

**Figure 1:**
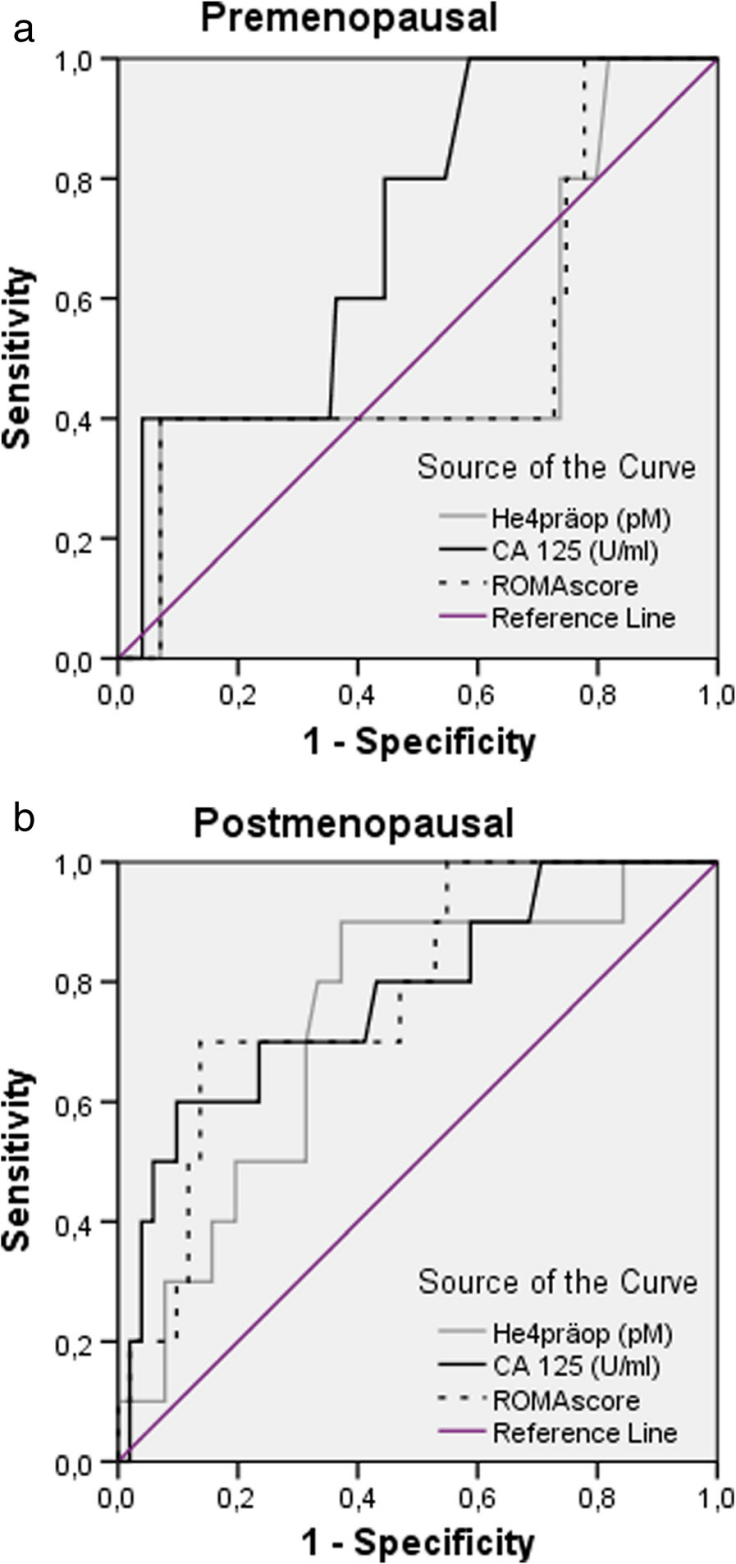
ROC curves for preoperative HE4 and CA125 concentrations and for ROMA scores in premenopausal (a) and postmenopausal (b) patients.

The ROC analysis showed that in premenopausal patients, CA125 alone is superior to HE4 and ROMA in discriminating patients with BOT with invasive implants from patients with benign diseases and BOT without invasive implants (Figure 1a).

### HE4 and CA125 serum concentrations in postmenopausal patients

The median preoperative serum HE4 concentrations of the three groups differed significantly (p = 0.007), with 50 pM (range: 27-241 pM) in benign diseases, 76.55 pM (range: 40-461 pM) and 68 pM (range: 41-334 pM) in BOT patients with and without invasive implants, respectively. HE4 concentrations in benign diseases differed significantly from those concentrations found in BOT with (p = 0.007) and without invasive implants (p = 0.025), respectively. In contrast, there was no significant difference (p = 0.418) in the HE4 concentrations between the two BOT subgroups.

In postmenopausal patients CA125 values were significantly different in the three groups of patients (p = 0.003). Median CA125 expression in serum was 18 U/ml (range: 5-334 U/ml) in benign diseases, 76.5 U/ml (range: 12–259 U/ml) and 31.5 U/ml (range: 9-134 U/ml) in BOT patients with and without invasive implants, respectively. CA125 levels in BOT patients with invasive implants were higher compared to BOT without invasive implants, but no statistical significance has been reached (p = 0.159).

The ROC analysis revealed that CA125 alone is slightly superior to HE4 and similar to ROMA in discriminating postmenopausal patients with BOT with invasive implants from patients with benign diseases and BOT without invasive implants. AUC for CA125, HE4 and ROMA were 0.778 (p = 0.006, 95% CI: 0.615-0.942), 0.732 (p = 0.021, 95% CI: 0.570-0.894) and 0.782 (p = 0.017, 95% CI: 0.637-0.927), respectively (Figure 1b).

Different cut-off values for CA125, HE4 and ROMA were analysed. The results for premenopausal and postmenopausal patients are presented in Table 2.

**Table 2:** Sensitivity and specificity for different cut-off values

**Biomarker**	**Cut-off value**	**Sensitivity (%)**	**Specificity (%)**
**Premenopausal**			
HE4	93 pM	50	92.9
CA125	18.5 U/ml	100	41.4
CA125	180 U/ml	40	96
ROMA	3.45%	100	22.2
ROMA	28.5%	40	92.9
***Postmenopausal***			
HE4	66 pM	90	62.7
HE4	79 pM	50	80.4
CA125	35 U/ml	60	90.2
CA125	60 U/ml	70	76.5
ROMA	16.2%	90	47.1
ROMA	34.9%	70	86.3
ROMABOT	8%	90	43.1
ROMABOT	16%	70	76.5

ROMA showed to be inferior to the single-marker use of CA125 in both pre- and postmenopausal patients. As a result of this finding, we established a new algorithm based on the ROMA by Moore et al. [19,20] using logistic regression with LN (CA125) and LN (HE4).

The ROC curve analysis revealed a greater AUC of 0.778 for ROMABOT compared to an AUC of 0.739 for ROMA. Since we could not reveal a superiority of ROMABOT compared to ROMA, we decided to only use ROMA for further analyses.

### HE4 and CA125 predictive values in matched samples from patients with benign diseases, BOT with and without invasive implants

The median age in all three groups, including both premenopausal and postmeno-pausal patients, showed significant differences (p = 0.022) with 48 years (range: 19–86 years) in benign diseases, 55 years (range: 25–87 years) and 44 years (range: 16–83 years) in BOT with and without invasive implants, respectively. Biomarkers usually can be expressed differently according to age. Therefore, and due to the low number of patients with invasive implants, we decided to match the BOT patients with invasive implants (14 out of 15, one was excluded since no matching pair was found) according to age and histology. Every patient was matched with one BOT patient without invasive implants and with 4 other patients with benign diseases. All analyses presented above were repeated within this setting.

Likewise the analysis of patients of all three groups, the matching process revealed significant results for HE4 concentrations for BOT with (p = 0.009) and without (p = 0.020) invasive implants compared to benign diseases. No significant difference (p = 0.822) between BOT with and without invasive implants was found. The CA125 concentration for BOT without invasive implants compared to benign diseases showed no significant difference (p = 0.087). CA125 concentration of BOT with invasive implants differed significantly (p = 0.001) compared to benign diseases. Similar to the median HE4 concentrations, there was no significant difference (p = 0.201) in CA125 concentrations between BOT with and without invasive implants.

The ROC curve analyses showed that CA125 is superior to HE4 and ROMA in discriminating patients with BOT with invasive implants from patients with benign diseases and BOT without invasive implants, even within this setting. AUC for CA125, HE4 and ROMA were 0.788 (p = 0.045, 95% CI: 0.598-0.978), 0.660 (p = 0.266, 95% CI: 0.387-0.933) and 0.744 (p = 0.090, 95% CI: 0.537-0.951), respectively.

In postmenopausal patients, CA125 performed slightly better than ROMA and both performed better than HE4 alone in discriminating borderline tumors from benign conditions, as well as to predict the presence of invasive implants. AUC for CA125, HE4 and ROMA were 0.762 (p = 0.14, 95% CI: 0.585-0.939), 0.723 (p = 0.36, 95% CI: 0.556-0.891) and 0.728 (p = 0.32, 95% CI: 0.561-0.896), respectively.

## Discussion

The aim of our study was to assess whether preoperatively measured serum concentrations of HE4 and CA125 alone or within ROMA score can detect the presence of borderline tumors of the ovary (BOT) or predict the presence of invasive implants. HE4 was detected using EIA(e) technology. Previous study showed no significant differences between performance of the HE4 kits and methods being used [21].

Current study showed that CA125 is superior in both pre- and postmenopausal patients to ROMA and both of them are superior to HE4 alone in discriminating borderline tumors from benign diseases. The introduction of ROMABOT as a new score for the prediction of invasive implants didn’t increase the performance of the test. Both CA125 and HE4 couldn’t predict the presence of invasive implants. To the best of our knowledge, this study is the first one investigating the role of HE4 and CA125 in predicting BOT with invasive and non-invasive implants.

The limitation of this study is the low number of BOT specimens, especially the BOT with invasive implants. The presence of invasive implants is a rare condition of the BOT. In the multicentric study of Du Bois et al. [12], invasive implants were seen in only 26 (2.7%) from 950 BOT patients. In our study, 15 patients were presenting invasive implants at the time of diagnosis. The study of Du Bois et al., has analyzed the largest cohort of BOT until now. The results of the study showed that tumor stages higher than FIGO I and presence of implants have been associated with increased risk for recurrence and malignant transformation. Nevertheless, there was no statistical significant impact of invasive vs. non-invasive implants, this maybe due to low number of patients having invasive implants. Within 950 BOT patients analysed, 152 (16.2%) presented peritoneal implants and 26 (2.7%) invasive implants [12].

The lack of early cancer biomarkers is currently a major obstacle for blood-based early detection, within ovarian cancer patients [22]. The quantity of biomarkers shed into the general circulation is depending on volume of the tumor, secretion rates from tumoral and healthy cells, tumor vascularization, vascular permeability, effects of cancer heterogeneity on tumor growth [22]. In a study of Hori et al., using a mathematical model, they aimed to quantify the time required for a growing malignant tumor cell population to reach a sufficient size so that its shed blood biomarker levels were high enough to be detected using current clinical blood biomarker assays. The results showed that a tumor could grow unnoticed for over 10.1 years when it will reach a volume corresponding to a spherical diameter of about 25.36 mm before becoming detectable by current clinical blood assays and by transvaginal ultrasound [22]. Borderline tumors are difficult masses to correctly diagnose preoperatively using ultrasound as their macroscopic features may overlap with both invasive and benign ovarian tumors [23]. For this reason BOTs are correctly classified before surgery in only 29%-69%. Until now there are no reliable imaging diagnostics for BOT [23].

Currents studies suggested that some of the BOT lesions might be possible precancerous condition that might evolve to low grade serous ovarian cancer [24-26]. HE4 and CA125 are mainly expressed by high grade serous and endometrioid carcinoma of the ovary [27,28].

Shih et al. analyzed the impact of histological and clinical features in the process of relapse in 266 patients with BOT. In this study 143 (53.8%) presented with elevated serum CA125 values (>35 IU/L). Patients with normal baseline CA125 levels had a significant longer 3-year PFS compared to patients with elevated CA125 levels at time of diagnosis [29]. The same study showed that preoperative elevated CA125 was associated with increase risk of recurrence in women with BOT.

Most authors conclude that CA125 concentrations in BOT patients are often negative or at least not significantly increased [30,31]. Du Bois et al. could not reveal elevated CA125 levels in 46.2% of 1937 patients diagnosed with a BOT [4] while a study by Gotlieb et al. showed increased CA125 concentrations prior surgery in 75% of BOT patients with serous histology. Only 30% of mucinous BOT showed elevated CA125 levels [32]. In a recent study by Nassir et al. no association between the HE4 tissue expression of 25 BOT without invasive implants and histological type, age, CA125 levels in blood and FIGO stage was found [33].

In the recent publication of Kaijser et al., the authors analysed the value of serum HE4 or ROMA score as second-stage tests for tumors thought to be difficult to characterize on the basis of ultrasound findings [34]. From 360 patients with pelvic tumors, examiners were highly confident in 54%, moderately confident in 38% and completely uncertain about their diagnosis in 8% of the cases. Most of the unclassifiable tumors were benign (79%) followed by BOT (14%). The sensitivity and specificity of subjective assessment was 67% and 70%, respectively. HE4 and ROMA had a poor discriminatory capacity. The conclusion of the study was that including ROMA and HE4 as second-test after transvaginal ultrasonography will lead to a decrease in overall test performance [34].

In our study HE4 and CA125 were significantly differently expressed in benign diseases vs. BOT and BOT with invasive implants only in postmenopausal patients. Nevertheless no biomarker could differentiate between the two subgroups of BOT.

Large population studies showed that both CA125 and HE4 vary with the age at diagnosis. Urban et al., showed that HE4 increases slowly with the age and particularly in women aged > =55 years, where increases in about 27.1% per decade were reported [35]. Therefore we performed an analysis where BOT patients with and without invasive implants were matched according to age and histology with benign diseases. Even this subset of analysis confirmed previous results.

Our study revealed that HE4 and ROMA are not better in predicting the presence of invasive implants in BOT than CA125 alone. The lack of sensitivity and specificity of HE4 and CA125 biomarkers in predicting the presence of BOT, underline once more that these tumors are a specific entity of ovarian tumors, which pathogenicity is not yet understood.

Due to small number of patients presenting with invasive implants, the long duration of follow-up needed for accurate evaluation and the lack of literature multicentric, international studies having BOT as main focus are needed. Serial HE4 and CA125 serum values similar to UKCTOCS trial [36] together with ultrasound criteria might increase the sensitivity and specificity of BOT diagnosis.

## Competing interests

The authors declare no competing interests.

## Authors’ contribution

EIB participated in study design, HE4 analysis, patients’ inclusion and article writing. TvanG carried out CA125 analysis and participated to the article reviewing. RR carried out the statistics and reviewed the article. MN documented the clinical data and drafted the manuscript. RC participated at article reviewing. KG participated at documentation of clinical data and article editing. Prof. DT participated at article reviewing and design. Prof. IV participated at article reviewing. Prof. JS was involved in study design and article reviewing. All authors read and approved the final manuscript.
